# Supplementary material for: Chlorophyll Inhibits the Digestion of Soybean Oil in Simulated Human Gastrointestinal System
Source: Nutrients. 2022 Apr 22;14(9):1749. doi: 10.3390/nu14091749 (PMC9101154; doi:10.3390/nu14091749)
Supplement: Supplementary file 1 [file nutrients-14-01749-s001.zip › nutrients-1644281-supplementary.pdf]

**Supplementary Table S1.**

The Stern-Volmer quenching constant ($K_{sv}$), bimolecular quenching rate constant ($K_q$), binding constants ($K_a$) and number of binding sites ($n$) for pheophytin binding to pancreatic lipase.

| | $K_{sv}$ (×$10^4$ L/mol) | $K_q$ (×$10^{12}$ L/(mol·s)) | $K_a$ (×$10^3$ L/mol) | $n$ |
|---|---|---|---|---|
| Phe-lipase | 0.2392 ± 0.0025 | 0.2392 ± 0.0025 | 0.1325 ± 0.0001 | 1.39562 ± 0.0135 |
